# Reactive Sulfur Species Produced by Cystathionine γ-lyase Function in the Establishment of *Mesorhizobium loti*–*Lotus japonicus* Symbiosis

**DOI:** 10.1264/jsme2.ME23021

**Published:** 2023-09-12

**Authors:** Mitsutaka Fukudome, Haruka Ishizaki, Yuta Shimokawa, Tomoko Mori, Nahoko Uchi-Fukudome, Kamolchanok Umnajkitikorn, Ei-ichi Murakami, Toshiki Uchiumi, Masayoshi Kawaguchi

**Affiliations:** 1 Faculty of Agriculture, Kagawa University, Miki-cho, Kita-gun, Kagawa 761–0795, Japan; 2 Division of Symbiotic Systems, National Institute for Basic Biology, 38 Nishigonaka, Myodaiji, Okazaki, Aichi 444–8585, Japan; 3 Graduate School of Science and Engineering, Kagoshima University, 1–21–35 Korimoto, Kagoshima 890–0065, Japan; 4 Trans-Omics Facility, Trans-Scale Biology Center, National Institute for Basic Biology, 38 Nishigonaka, Myodaiji, Okazaki, Aichi 444–8585, Japan; 5 Graduate School of Medical and Dental Sciences, Kagoshima University, 8–35–1 Sakuragaoka, Kagoshima 890–8544, Japan; 6 School of Crop Production Technology, Institute of Agricultural Technology, Suranaree University of Technology, Nakhon Ratchasima 30000, Thailand; 7 Nihon Pall Ltd. Scientific and Laboratory Service, Ami-machi, Inashiki-gun, Ibaraki 300–0315, Japan

**Keywords:** root nodule symbiosis, reactive sulfur species, cystathionine γ-lyase, sulfur metabolism, sulfane sulfur

## Abstract

Reactive sulfur species (RSS) are present in root nodules; however, their role in symbiosis and the mechanisms underlying their production remain unclear. We herein investigated whether RSS produced by the cystathionine γ-lyase (CSE) of microsymbionts are involved in root nodule symbiosis. A *cse* mutant of *Mesorhizobium loti* exhibited the decreased production of hydrogen sulfide and other RSS. Although the *CSE* mutation of *M. loti* did not affect the early stages of symbiosis, *i.e.*, infection and nodulation, with *Lotus japonicus*, it reduced the nitrogenase activity of nodules and induced their early senescence. Additionally, changes in the production of sulfur compounds and an increase in reactive oxygen species (ROS) were observed in the infected cells of nodules induced by the *cse* mutants. The effects of CSE inhibitors in the *L. japonicus* rhizosphere on symbiosis with *M. loti* were also investigated. All three CSE inhibitors suppressed infection and nodulation by *M. loti* concomitant with decreased RSS levels and increased ROS and nitric oxide levels. Therefore, RSS derived from the CSE activity of both the microsymbiont and host plant are required for symbiosis, but function at different stages of symbiosis, possibly with crosstalk with other reactive mole­cular species.

Leguminous plants obtain and use atmospheric dinitrogen as nitrogen nutrition via root nodule symbiosis with rhizobia. Various nutrient molecules, including sulfur, are involved in establishing this symbiosis. In pea (*Pisum sativum* L.) grown in sulfur-deficient soil, nitrogen-fixation activity, plant growth, and yield were reduced, and these phenotypes were improved by supplying large amounts of sulfur (Anderson and Spencer, 1950). In white clover (*Trifolium repens*), sulfur deficiency was shown to affect nodule numbers, nitrogen-fixation activity, and amino acid metabolism in the nodules (Varin *et al.*, 2010). In *Lotus japonicus*, the sulfate transporter Sst1 is specifically expressed in the root nodules, and *sst1* mutant lines were found to exhibit significantly reduced nitrogen-fixation activity (Krusell *et al.*, 2005). Sulfur is required to construct the molecules that are essential for symbiosis, including nitrogenase (Rubio and Ludden, 2008) and ferredoxin (Carter *et al.*, 1980), and has been associated with ferredoxin and leghemoglobin levels in nodules (Scherer *et al.*, 2008). However, the roles of sulfur in root nodule symbiosis remain unclear.

Reactive sulfur species (RSS), including sulfane sulfur and hydrogen sulfide (H_2_S), function as signal molecules and reductants *in vivo* (Gruhlke and Slusarenko, 2012). Among RSS, the function of H_2_S has been extensively examined, in contrast to sulfane sulfur, which has received little attention. Sulfane sulfur, a divalent sulfur atom bonded to another sulfur, *e.g.*, a persulfide or polysulfide, exhibits high antioxidant activity, and its activity and role have primarily been investigated in mammals and bacteria (Gruhlke and Slusarenko, 2012). In plants, H_2_S is involved in signal transduction in various physiological processes (Zhang *et al.*, 2009; Scuffi *et al.*, 2014; Laureano-Marín *et al.*, 2016; Li *et al.*, 2016; Aroca *et al.*, 2018), and sulfane sulfur also plays a role in plant physiology (Gruhlke and Slusarenko, 2012; Yamasaki *et al.*, 2019). However, studies on H_2_S and sulfane sulfur in root nodule symbiosis between legumes and rhizobia are not as abundant as in general plant physiology studies. Some studies indicated that RSS are involved in root nodule symbioses. For example, H_2_S donors in soybean were found to promote root nodule symbiosis, which increased plant growth and nitrogenase activity (Zou *et al.*, 2019). In *L. japonicus*, sulfane sulfur localizes to infection threads (ITs) in the root hair and infected cells in nodules, and its crosstalk with H_2_S, reactive oxygen species (ROS), and reactive nitrogen species (RNS) is considered to be involved in the establishment of symbiosis (Fukudome *et al.*, 2020).

Sulfane sulfur is produced by enzymes, such as cystathionine γ-lyase (CSE), cystathionine β-synthase (CBS), and 3-mercaptopyruvate sulfur transferase (reviewed by Walsh and Giedroc, 2020). The CSE of rhizobia produces H_2_S in the root nodules of soybean, with H_2_S serving as an antioxidant element that is essential for nitrogen fixation (Zou *et al.*, 2020). In addition, mutations in a CBS-like domain-containing protein of *Medicago truncatula* acting as the host plant resulted in abnormal root nodule symbiosis (Sinharoy *et al.*, 2016). Collectively, these studies support the notion that RSS produced by rhizobia and host plants are crucial for symbiosis. Although CSE is involved in the production of sulfane sulfur and H_2_S (Ida *et al.*, 2014), to the best of our knowledge, the production of sulfane sulfur by CSE in root nodule symbiosis has not yet been investigated.

In the present study, we investigated whether CSE of *M. loti*, a microsymbiont of *L. japonicus*, is involved in the production of sulfane sulfur during root nodule symbiosis. We also examined the effects of CSE on H_2_S, ROS, and RNS levels in nodules. The effects of rhizobial CSE were analyzed by evaluating sulfur and amino acid metabolism in symbiosis with *CSE*-disrupted mutants. The effects of CSE inhibitors in the *L. japonicus* rhizosphere on RSS production and root nodule symbiosis were also assessed. The inhibition of CSE activity by an exogenous inhibitor resulted in a different symbiotic phenotype than that caused by rhizobial *cse* mutants. Overall, these results indicate that sulfur metabolism in both the microsymbiont and host plant may be involved in the establishment of symbiosis.

## Materials and Methods

### Biological materials and plant growth conditions

Plants of the *L. japonicus* accession Gifu B-129 line were germinated and grown as previously described (Nagata *et al.*, 2008). Briefly, 5 days after germination, seedlings were transplanted to‍ ‍autoclaved vermiculite containing B&D medium (Broughton and Dilworth, 1971) and inoculated with a cell suspension (10^8^‍ ‍cells‍ ‍mL^–1^ in water) of *M. loti* strain MAFF303099 (Kaneko *et al.*, 2000) and its signature-tagged mutagenesis (STM) strains. Plants were grown under controlled conditions with a photosynthetically active radiation level of 150‍ ‍μmol photons m^–2^ s^–1^ (16-h photoperiod) at 25°C for 4, 6, and 8‍ ‍weeks post-inoculation (wpi). In MAFF303099, the *CSE* gene is encoded on two open reading frames, mlr1566 and mll4503. Among them, mll4503 gas been predicted by MicrobesOnline (http://www.microbesonline.org/operons/gnc266835.html) to be on the same operon as *CBS* (mll4505), which functions cooperatively with CSE in RSS production. Therefore, in this study, we focused on the *CSE* encoded by mll4503, which is predicted to be closely involved in the production of RSS as a “*CSE* gene”. STM strains of *M. loti* carry a Tn5-based mini-transposon, randomly distributed throughout the genome (Shimoda *et al.*, 2008). In this mutagenesis strategy, each STM strain has one mutation. Three STM strains (clone IDs: 12T02e02, 14T06a12, and 20T03b07) with a mini-transposon inserted into mll4503, an open reading frame encoding CSE, were designated *cse1*, *cse2*, and *cse3*, respectively. The *CSE* gene was 1,185 bp, and *cse1*, *cse2*, and *cse3* had Tn5-based mini-transposons inserted at positions 609, 298, and 304 bp, respectively (Supplementary Fig. S1).

### Bacterial growth conditions and resistance assay

When the optical density at 600‍ ‍nm (OD_600_) of the culture was 0.4–0.5, cells were harvested, washed twice with yeast mannitol (YM) medium (Wacek and Brill, 1976), and suspended in YM medium to achieve an OD_600_ of 0.2. Hydrogen peroxide (H_2_O_2_) was used to elicit oxidative stress; it was added to the bacterial suspension to produce final concentrations of 3, 6, and 9‍ ‍mM, and the suspensions were incubated with shaking at 26°C for 24 h. Sodium cyanide (NaCN) as a sulfane sulfur scavenger was added to the bacterial suspension to produce final concentrations of 5, 10, 50, and 100‍ ‍μM, and the suspensions were incubated with shaking at 26°C for 16 h. DL-propargylglycine (PAG), β-cyanoalanine (BCA), D-penicillamine (D-pen) (as CSE inhibitors), and hypotaurine (HT) (as a H_2_S scavenger) were added to bacterial suspensions to produce final concentrations of 100‍ ‍μM, and the suspensions were incubated with shaking at 26°C for 16 h. OD_600_ was measured using Miniphoto518R (Taitec).

### Nitrogenase activity

The nitrogenase activity of nodules was assessed by measuring acetylene reduction activity (ARA) according to the method reported by Shimoda *et al.* (2009). Whole plants were placed in glass tubes containing wet filter paper, and the tubes were filled with an acetylene and air mixture (C_2_H_2_:air=1:9‍ ‍[v/v]). After a 1-h incubation at 25°C, the level of ethylene in the gas phase was evaluated using a GC-8A gas chromatograph (Shimadzu).

### Sectioning of root nodules

To create agar sections of nodules, nodules were embedded in 5% agar and sectioned to a thickness of 70‍ ‍μm using a vibratome. To create resin sections of nodules, nodules were fixed with 4.0% paraformaldehyde and 2.5% glutaraldehyde in 0.1 M sodium phosphate buffer (pH 7.2) at 4°C overnight. Fixed samples were then dehydrated using a graded ethanol series, embedded in JB4 resin (Polysciences), and sectioned to a thickness of 3‍ ‍μm. Nodule sections were stained with 0.5% (w/v) toluidine blue reagent according to the manufacturer’s instructions.

### Detection of endogenous signal molecules produced in roots and nodules

The production of signal molecules in roots and nodules was detected and measured as previously described (Fukudome *et al.*, 2020). Briefly, the roots and agar sections of nodules were soaked for 1‍ ‍h with specific probes for each molecule as follows. Probes were dissolved in phosphate-buffered saline containing 137‍ ‍mM NaCl, 2.7‍ ‍mM KCl, 8‍ ‍mM Na_2_HPO_4_, and 2‍ ‍mM NaH_2_PO_4_ (pH 7.4). To detect sulfane sulfur, the roots and nodules were soaked in 10‍ ‍μM SSP4 (Dojindo) with 0.5‍ ‍mM cetyltrimethylammonium bromide. SSP4 has no membrane permeability, but may penetrate cell membranes in combination with cetyltrimethylammonium bromide. SSP4 reacts with sulfane sulfur and with the polysulfides of glutathione and cysteine. To detect NO, samples were soaked in 20‍ ‍μM DAF-FM DA (GORYO Chemical). DAF-FM DA has membrane permeability and is deacetylated to DAF-FM by esterase inside the cell, wherein DAF-FM reacts with the endogenous NO oxidation product N_2_O_3_ to form a highly fluorescent triazole. To detect ROS, samples were soaked in 10‍ ‍μM CellROX Green Reagent (Invitrogen). The cell-permeable CellROX reagent is essentially non-fluorescent in its reduced state, but exhibits a strong fluorescent signal upon oxidation. To detect H_2_S, samples were soaked in 5‍ ‍μM Hsip-1 DA (Dojindo). Hsip-1 DA has membrane permeability. Through esterase activity inside the cell, Hsip-1 DA is deacetylated to Hsip-1, which is a fluorescent molecule (a copper [II] ion chelate type) that specifically reacts with H_2_S. In the roots, the endogenous production of each signal molecule was detected via fluorescence or stereofluorescence microscopy. Fluorescence images were captured using an A1si-90i microscope and epifluorescence images with an Eclipse 90i microscope (both from Nikon). In all cases, fluorescence intensity was quantified using Image J (Version 1.51; NIH, Bethesda, MD, USA).

### Detection of endogenous signal molecule production in *M. loti* cells

Sulfane sulfur, H_2_S, and ROS production in *M. loti* cells was assessed using SSP4, Hsip-1 DA, and CellROX Green Reagent, respectively. Briefly, SSP4, Hsip-1 DA, and CellROX Green Reagent were added together to the cell suspension to produce a final concentration of 10‍ ‍μM for each chemical. The fluorescent intensity ratio was analyzed using an e-spect2 (Malcom).

### Analysis of sulfur metabolomics and amino acid profiling

Freshly collected samples were frozen and stored at –80°C until used. A sulfur metabolomic ana­lysis was performed by the Sulfur Index service in Japan using liquid chromatography coupled with tandem mass spectrometry (LC–MS/MS) as previously described (Kawano *et al.*, 2015; Tanaka *et al.*, 2019; Takewaki *et al.*, 2020). Briefly, sulfur-containing compounds in the samples were extracted by adding methanol and converted to fluorescent derivatives using monobromobimane. Target metabolite levels were measured from the peak area using mass chromatography and represented as relative amounts after normalization against the peak area of the internal standard (D-camphor-10-sulfonic acid). Regarding amino acid profiling, tissue samples were collected using the method applied for the Sulfur Index service–based sulfur metabolomics ana­lysis and homogenized using a Multi-Beads Shocker (YASUI KIKAI). Metabolites were extracted using 80% methanol containing 0.1% formic acid and internal standards (10‍ ‍pM ^13^C^15^N L-valine). The supernatant was collected after centrifugation at 13,000‍ ‍rpm and 4°C for 3‍ ‍min and then evaporated in a centrifugal concentrator. The residue was dissolved in 20‍ ‍μL of 0.1% formic acid and subjected to a LC–MS/MS ana­lysis using a Triple TOF 5600 system (AB Sciex).

## Results

### RSS production, ROS sensitivity, and amino acid metabolism of *cse* mutants

**Fig. 1.**
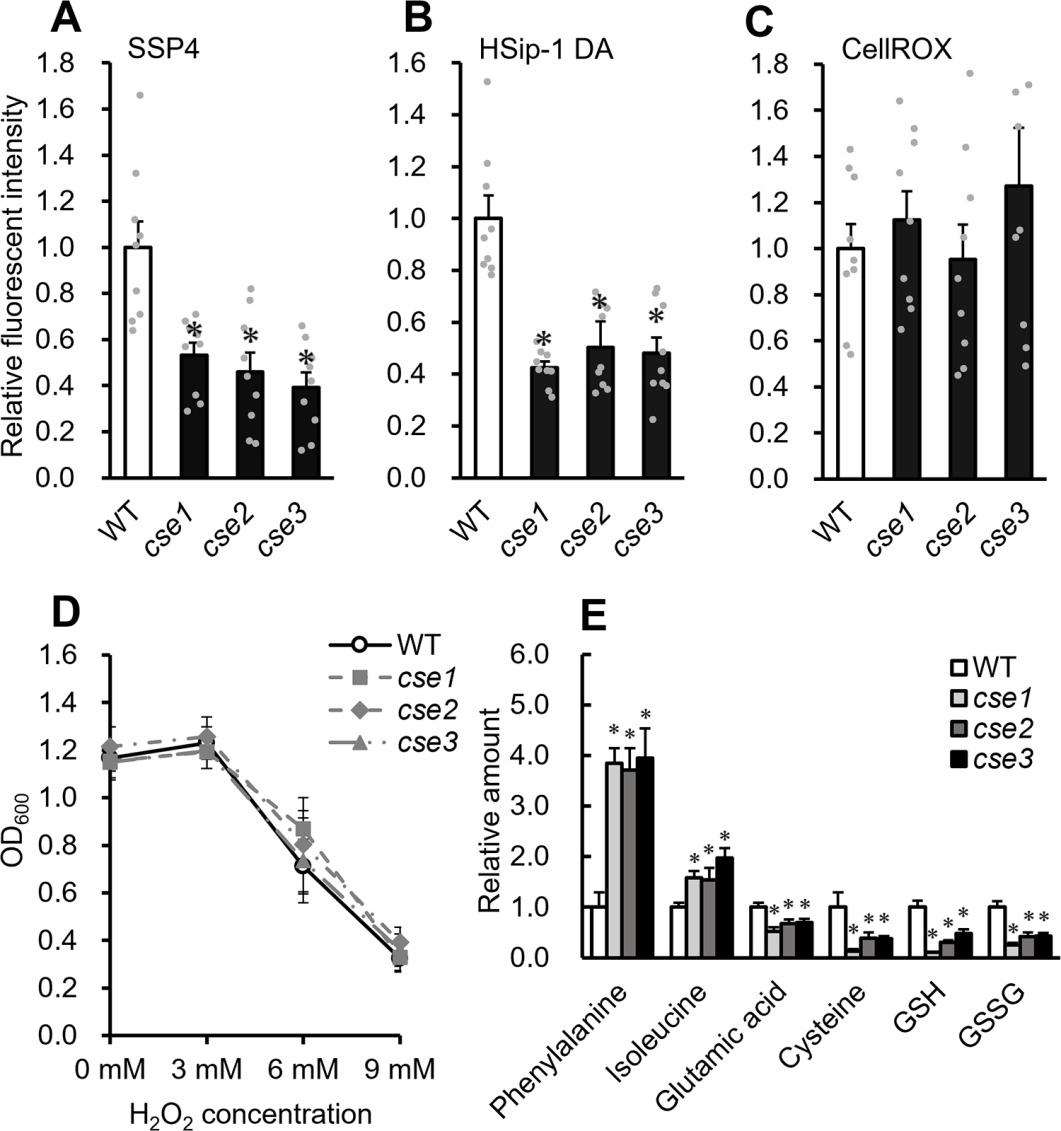
Characteristics of *cse* mutants under free-living conditions. (A, B, and C) Relative levels of sulfane sulfur, H_2_S, and ROS. Relative fluorescence intensity of (A) SSP4, (B) HSip-1 DA, and (C) CellROX in the cell suspension. (A, B, and C) Data are means±SE (*n*=9). (D) *Mesorhizobium loti* WT cells (solid line) or *cse* mutant cells (gray lines) were cultured in the presence of 3, 6, or 9‍ ‍mM H_2_O_2_. Cell density at OD_600_ was monitored. (E) Amino acid levels in bacterial cells. Amino acids were measured using LC–MS/MS. Data are means±SE (*n*=3). * *P*<0.05 according to the Student’s *t*-test.

The production of sulfane sulfur and H_2_S under free-living conditions was compared between wild-type (WT) *M. loti* strain MAFF303099 (Kaneko *et al.*, 2000) and the *CSE* gene–disrupted mutants *cse1*, *cse2*, and *cse3*. Sulfane sulfur and H_2_S production were measured according to fluorescence intensity using specific fluorescent probes: SSP4 and HSip-1 DA, respectively. The fluorescence intensities of SSP4 and HSip-1 DA were lower in the *cse* mutant suspension than in the WT suspension, indicating the lower production of sulfane sulfur and H_2_S in the mutants (Fig. 1A and B). Since RSS exhibit strong antioxidant activity, the effects of reduced sulfane sulfur and H_2_S production on endogenous ROS levels and sensitivity to oxidative stress in the mutants were investigated. ROS levels in the bacterial culture were measured using the specific fluorescent probe CellROX, and the detected levels of ROS did not significantly differ among the strains (Fig. 1C). Sensitivity to oxidative stress was evaluated according to growth in liquid medium supplemented with H_2_O_2_, which was added to the bacterial cultures in YM liquid medium at concentrations of 0, 3, 6, and 9‍ ‍mM. Sixteen hours after the addition of H_2_O_2_, the turbidity of *cse* mutants and WT was measured and did not significantly differ at any H_2_O_2_ concentration, indicating that sensitivity to oxidative stress did not significantly differ among strains (Fig. 1D). In addition, no significant differences were observed in the growth of the WT and *cse* mutants without oxidative stress (0‍ ‍mM H_2_O_2_ in Fig. 1D).

The effects of CSE mutations on metabolism were investigated. Twenty-eight simultaneously measurable amino acids (Supplementary Table S1) were detected, including sulfur-containing amino acids, such as cystine, cystathionine, and cysteine. *cse* mutants exhibited low levels of cysteine and glutathione, indicating abnormal sulfur metabolism. The mutants also exhibited low or high levels of several non-sulfur-containing amino acids, including phenylalanine, isoleucine, and glutamic acid (Fig. 1E).

### Effects of CSE gene mutations in *M. loti* on symbiosis

**Fig. 2.**
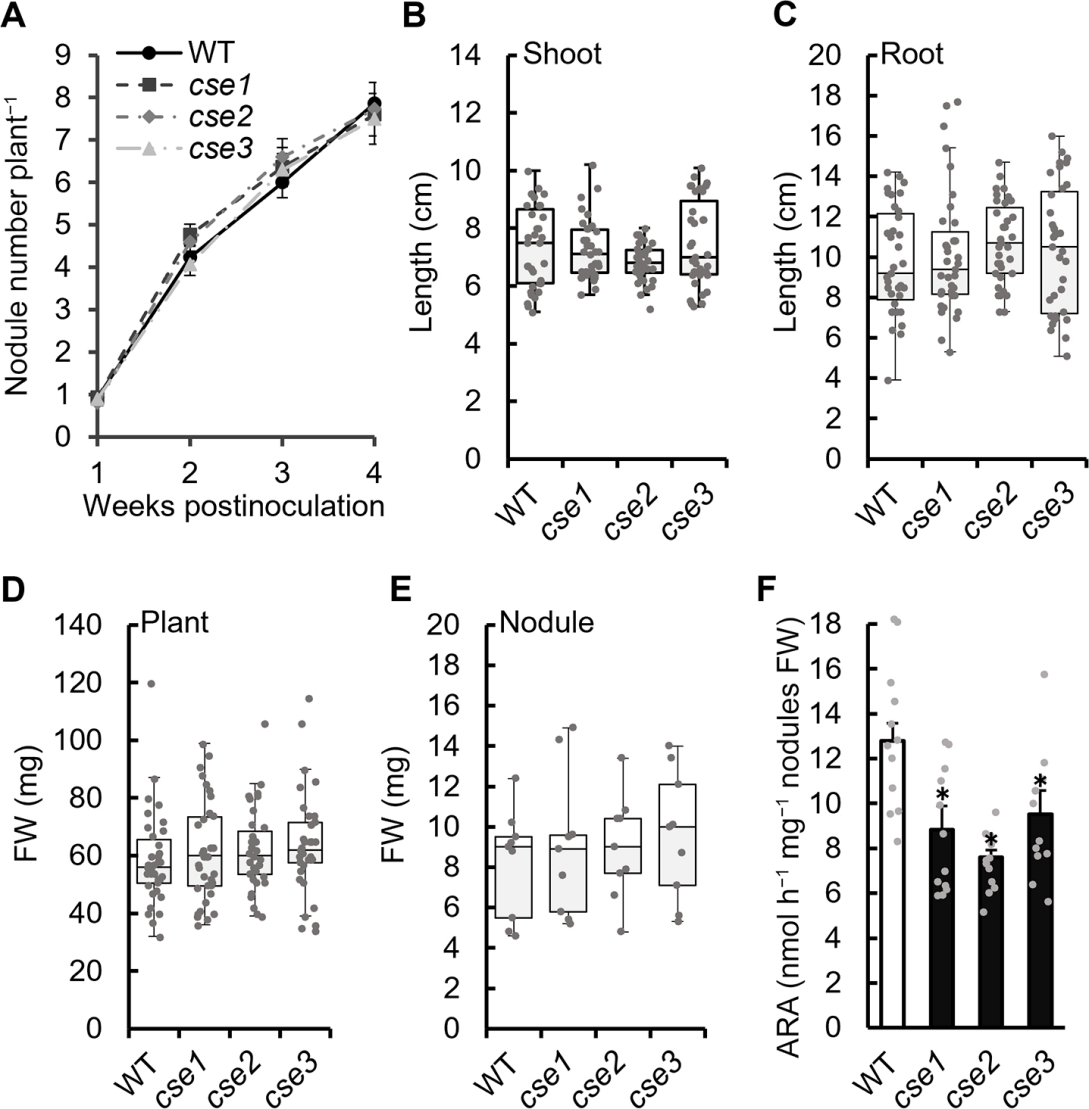
Symbiotic phenotypes of plants inoculated with *cse* mutants at 4‍ ‍weeks post-inoculation. (A) Number of nodules up to 1–4‍ ‍weeks post-inoculation (*n*=36). (B) Shoot and (C) root lengths (*n*=36). Fresh weight (FW) of (D) plants (*n*=36) and (E) root nodules (*n*=9). (F) ARA was measured using gas chromatography and is expressed as activity nodule^–1^ FW. Data are means±SE (*n*=12). * *P*<0.05 according to the Student’s *t*-test.

The effects of *CSE* gene mutations in *M. loti* on root nodule symbiosis with *L. japonicus* were investigated. *L. japonicus* was inoculated with each strain, and the number of nodules was counted at 1–4‍ ‍wpi. The number of nodules did not significantly differ between the WT and *cse* mutants at any time point (Fig. 2A). Plant growth (Fig. 2B, C, D, and E) and nodule appearance (Supplementary Fig. S2A) at 4‍ ‍wpi did not significantly differ between the WT and *cse* mutants; however, the nitrogenase activity of all three mutants was lower than that of WT (Fig. 2F).

**Fig. 3.**
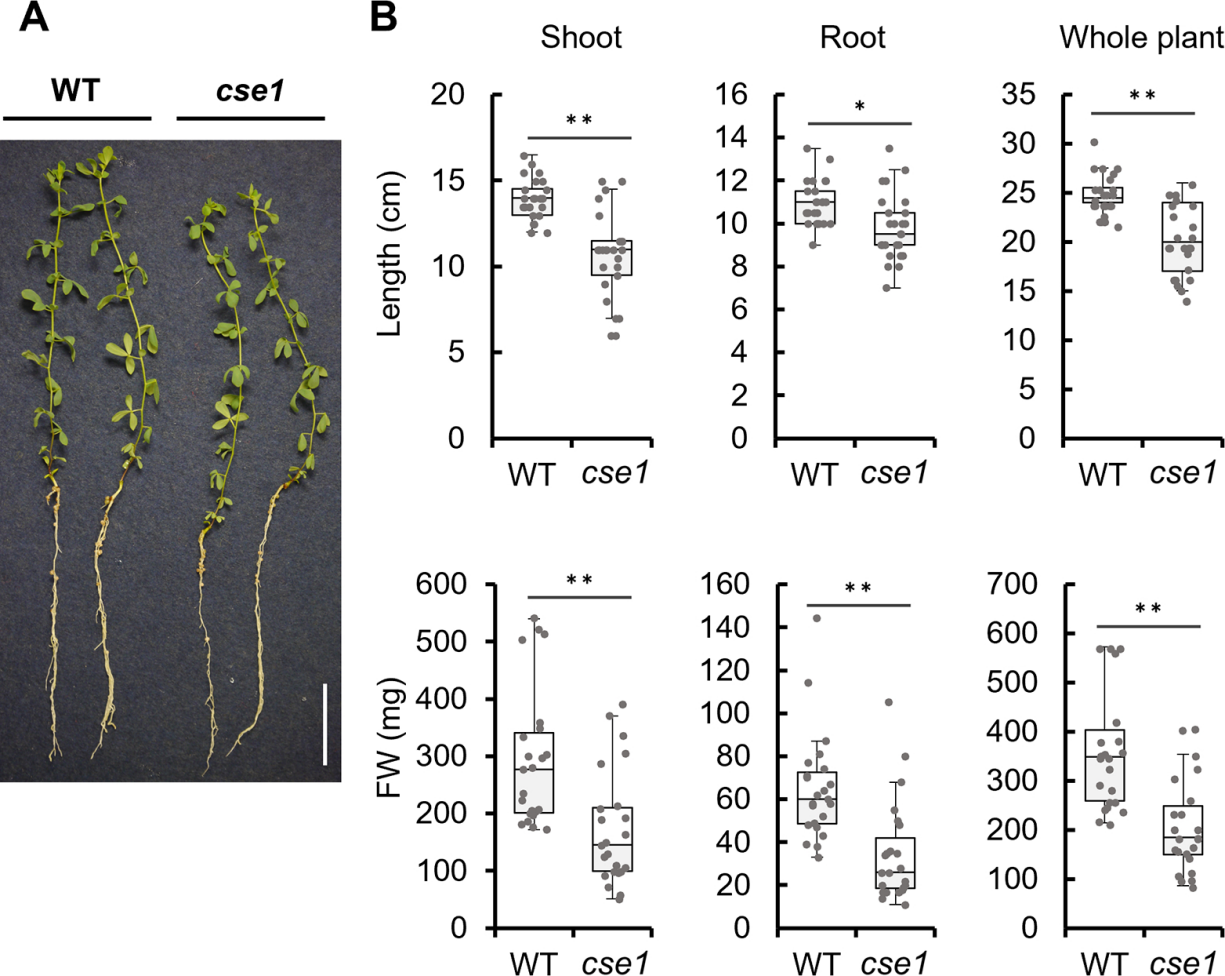
Symbiotic phenotype of plants inoculated with *cse* mutants at 8‍ ‍weeks post-inoculation. (A) Image showing plant growth. Scale bar: 3‍ ‍cm. (B) Lengths and fresh weights (FW) of the shoot, root, and whole plant (*n*=24). * *P*<0.05 and ** *P*<0.01 vs. the control (the Student’s *t*-test).

At 8 wpi, plants inoculated with *cse1* exhibited lower plant lengths and fresh weights than those inoculated with WT (Fig. 3A and B). Although the number of nodules did not significantly differ, the ratio of green nodules to total nodules was higher in plants inoculated with *cse1*, indicating earlier nodule senescence (Supplementary Fig. S2B). Additionally, the weight of nodules per plant was lower in plants inoculated with *cse1* than in those inoculated with WT (Supplementary Fig. S2C). Plant growth, the ratio of green nodules, and the nodule weight of plants inoculated with *cse2* and *cse3* at 8 wpi were similar to those of plants inoculated with *cse1* (data not shown).

**Fig. 4.**
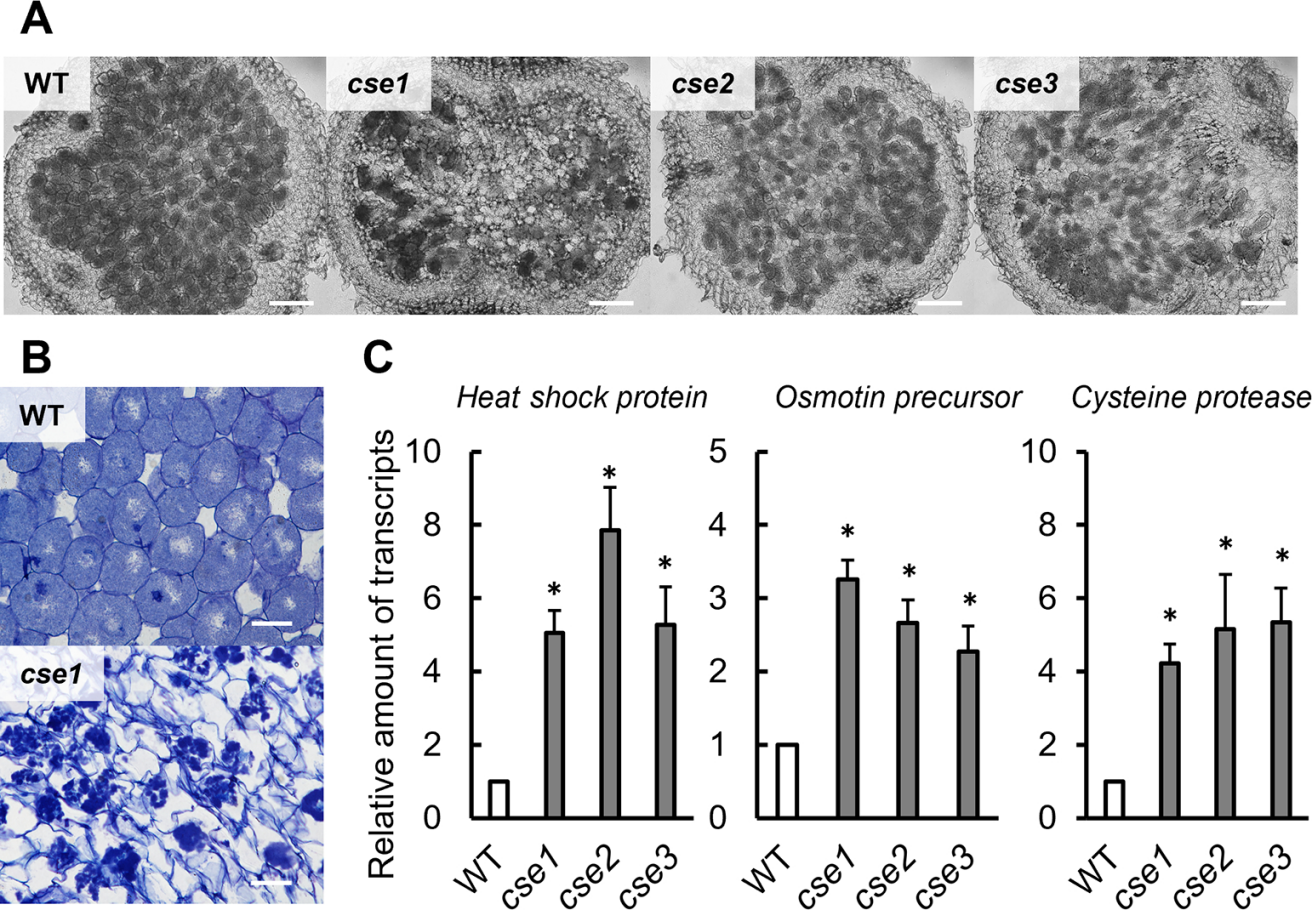
Light microscopy observation of 4-week-old nodules and the expression of senescence-related genes in these nodules. (A) Agar sections of nodules. Scale bars: 100‍ ‍μm. (B) Resin-embedded sections of nodules stained with toluidine blue. Scale bars: 20‍ ‍μm. (C) Expression of senescence-associated genes in the nodules. In each gene, mRNA expression levels in WT nodules were set to 1. Values are the means±SE of five biological replicates, each with three technical replicates. * *P*<0.05 vs. WT (the Student’s *t*-test).

Nodules induced by the *cse* mutants were examined histologically to investigate nodule senescence. At 5 wpi (*i.e.*, nodules aged 4‍ ‍weeks), many infected cells remained normal in the nodules of plants inoculated with WT, whereas infected cells were partially disintegrated in the nodules of plants inoculated with the *cse* mutants (Fig. 4A). Observations of disintegrated infected cells at a high magnification revealed the agglutination of rhizobia within the host cells (Fig. 4B). The expression of the senescence-related genes *heat shock protein*, *osmotin precursor*, and *cysteine protease Cyp2* was also examined using 4-week-old nodules. The expression of these senescence-related genes was higher in the nodules of plants inoculated with the *cse* mutants than in those of plants inoculated with WT (Fig. 4C).

### Sulfur and amino acid metabolism in root nodules induced by cse mutants

**Fig. 5.**
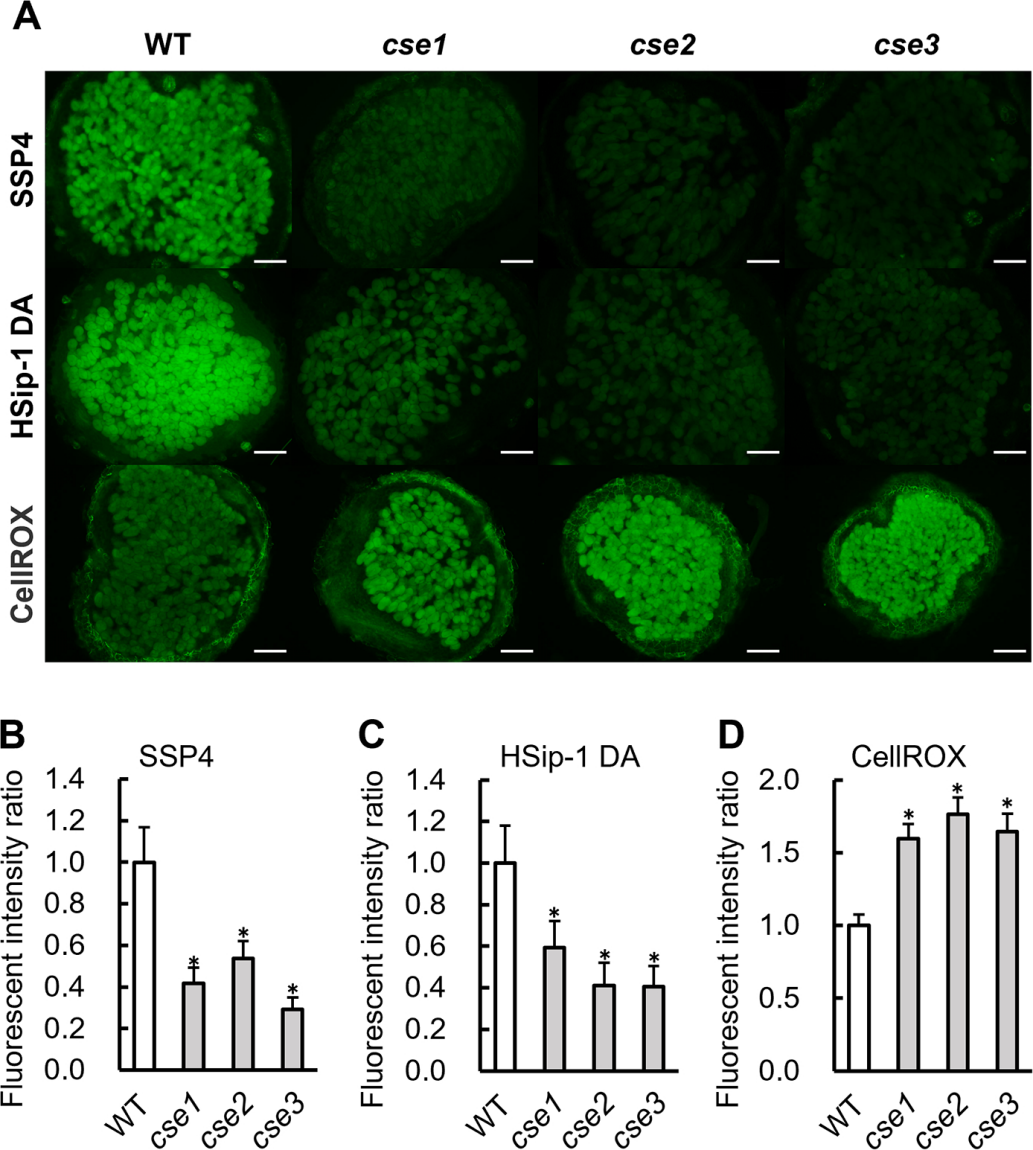
Reactive mole­cular species in root nodules. (A) Fluorescence microscopy images of WT- or *cse* mutant–infected nodules incubated with SSP4, HSip-1 DA, and CellROX. Scale bars: 100‍ ‍μm. (B, C, and D) Quantification of fluorescence intensity in SSP4 (B), HSip-1 DA (C), and CellROX (D) images. In (B, C, and D), values are the means±SE of nine biological replicates, each with three sections. * *P*<0.05 and ** *P*<0.01 vs. the control (the Student’s *t*-test).

We compared the levels of sulfane sulfur, H_2_S, and ROS in infected cells of the nodules of plants inoculated with the WT and *cse* mutants. Each infected cell was labeled with specific fluorescent probes, *i.e.*, SSP4, HSip-1 DA, and CellROX, and the detected fluorescence intensities were compared (Fig. 5A, B, C, and D). In the nodules produced by the *cse* mutants, the fluorescence intensity of SSP4 was lower than that in the nodules produced by WT, indicating a lower level of sulfane sulfur in infected cells (Fig. 5A and B). Similarly, the fluorescence intensity of HSip-1 DA was lower in the infected cells of nodules from plants inoculated with the *cse* mutants, indicating lower H_2_S levels in infected cells (Fig. 5A and C). In contrast, the fluorescence intensity of CellROX was higher in the infected cells of nodules from plants inoculated with the *cse* mutants, indicating higher levels of ROS (Fig. 5A and D). The level of nitric oxide (NO), which is involved in crosstalk with ROS, H_2_S, and sulfane sulfur in infected cells was assessed using the specific fluorescent probe DAF; no significant difference was observed in NO levels between the *cse* mutants and WT (Supplementary Fig. S3A and B).

**Fig. 6.**
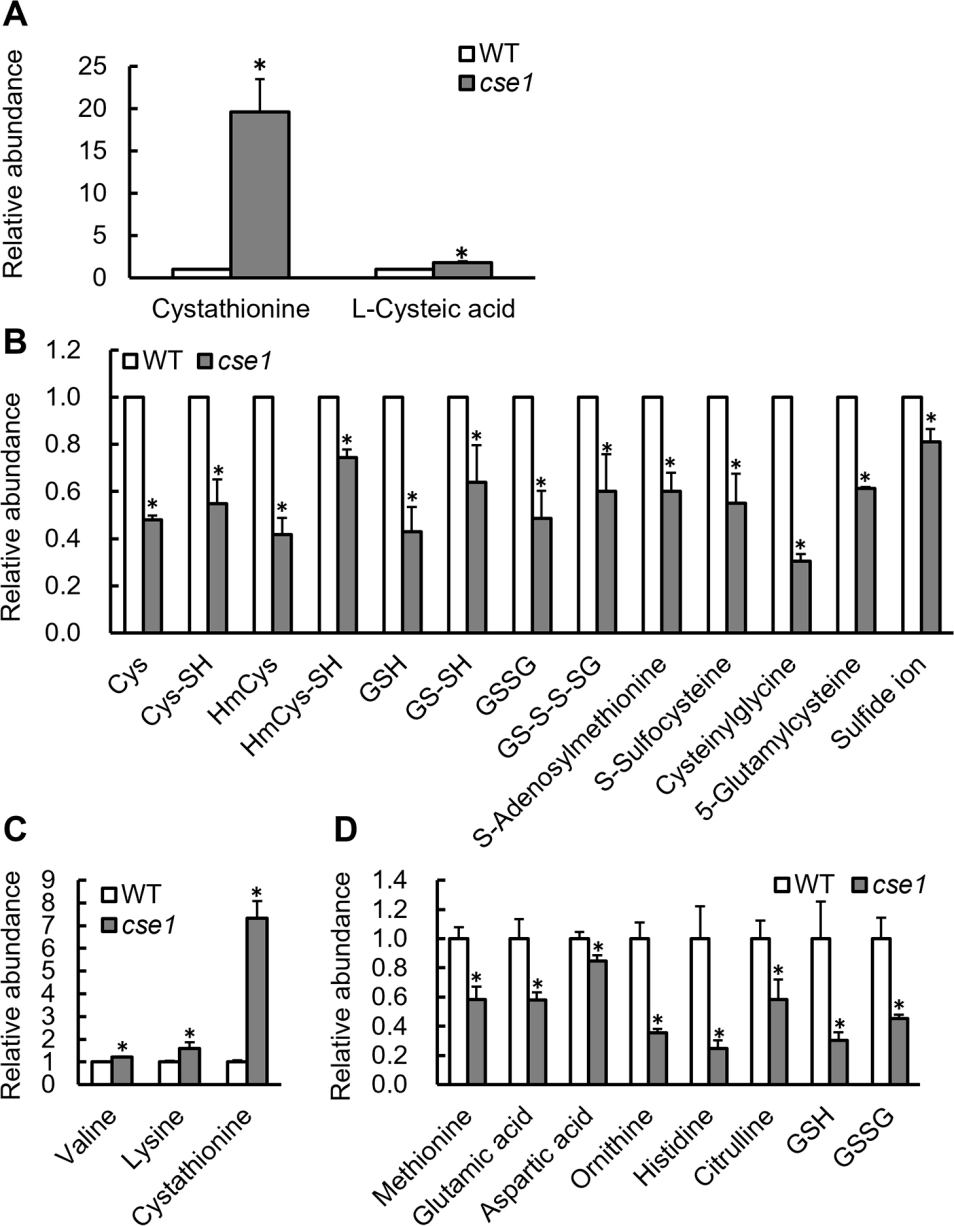
Sulfur compound and amino acid levels in nodules. (A) Sulfur compound levels were increased by the *cse* mutant. (B) Sulfur compound levels were decreased by the *cse* mutant. (A, B) Sulfur compound levels were measured using the sulfur index method, and data are expressed as the relative abundance ratio (*n*=3). (C) Amino acid levels were increased by the *cse* mutant. (D) Amino acid levels were decreased by the *cse* mutant. Amino acid levels were measured using LC–MS/MS, and data are expressed as the relative abundance ratio. (A, B, C, and D) Data are means±SE (*n*=7). * *P*<0.05 according to the Student’s *t*-test.

Since CSE is involved in sulfur metabolism and RSS production, we analyzed sulfur metabolites in the nodules induced by *cse* mutants. Sixty-eight sulfur compounds were measured using the sulfur index method, and 28 compounds were detected (Supplementary Table S2). Among these compounds, cysteine persulfide (CysSSH), homocysteine persulfide, and glutathione persulfide (GSSH), the glutathione trisulfide (GSSSH), and their oxides GSSSG and GSSSSG were identified as sulfane sulfur. In comparisons with the nodules induced by WT, the levels of 2 and 13 compounds increased (Fig. 6A) and decreased (Fig. 6B), respectively, in the nodules induced by *cse1*, whereas the levels of the 13 other compounds did not significantly change. The largest change in sulfur compound levels due to a *CSE* mutation was a 20-fold increase in cystathionine (Fig. 6A), indicating that *CSE* in *M. loti* contributed to cystathionine metabolism in the root nodules. The sulfur compounds that decreased were mainly molecules related to cysteine metabolism, such as S-sulfocysteine, cysteinylglycine, 5-glutamylcysteine, cysteine, homocysteine, and glutathione (Fig. 6B). The amino acid content was analyzed to assess the effects of *CSE* mutations on overall metabolism. Consistent with sulfur index method results, an increase in cystathionine (Fig. 6C) and a decrease in glutathione (Fig. 6D) were detected in the nodules induced by the *cse* mutants. In addition, *M. loti* *CSE* mutations affected amino acid metabolism in the nodules, with higher levels of valine and lysine (Fig. 6C) and lower levels of methionine, glutamic acid, aspartic acid, ornithine, histidine, and citrulline (Fig. 6D) than those found in nodules induced by WT.

### Effects of CSE inhibitors on the infection and symbiotic nitrogen fixation of rhizobia

**Fig. 7.**
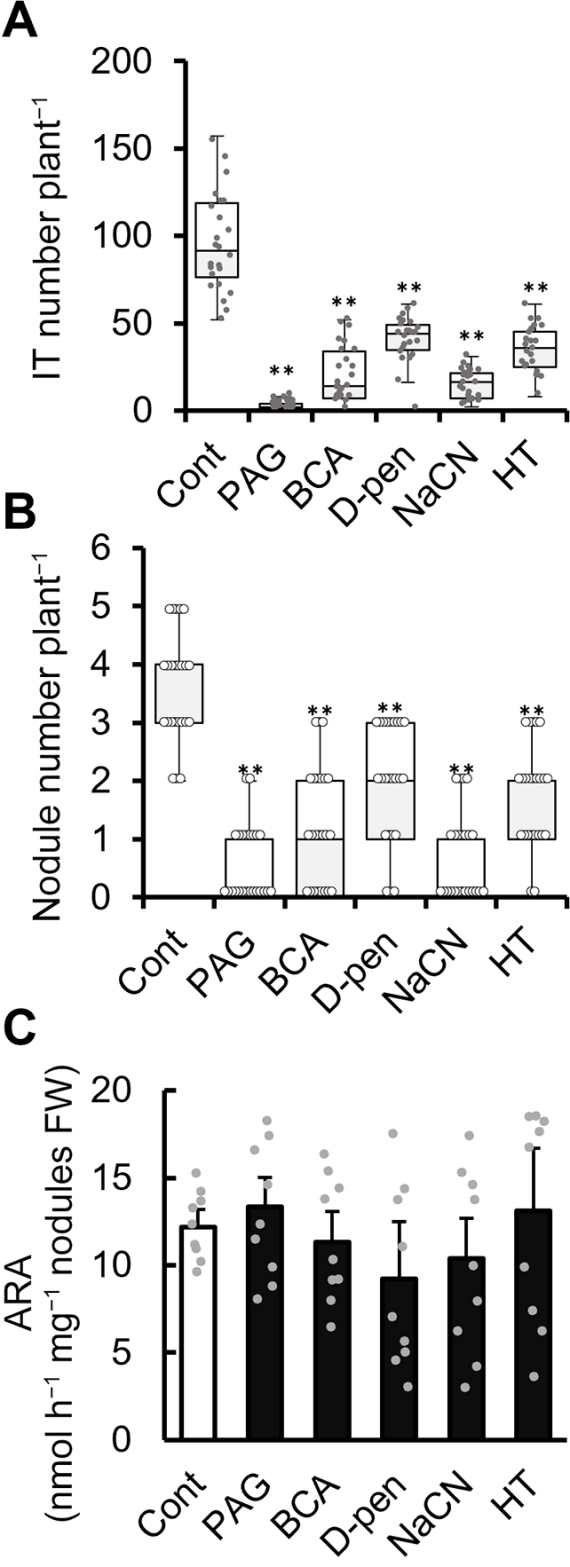
Effects of CSE inhibitors on root nodule symbiosis. PAG, BCA, and D-pen were used as CSE inhibitors; NaCN and HT were used as sulfane sulfur and H_2_S scavengers, respectively. (A) The number of infection threads (ITs) after 10 days and (B) the number of nodules at 4‍ ‍weeks post-inoculation with DsRed-labeled *Mesorhizobium loti* and treatment with each reagent (*n*=24). BCA, D-pen, and HT were used at a final concentration of 100‍ ‍μM for measuring the number of‍ ‍ITs and nodules, while PAG and NaCN were used at final concentrations of 50 and 10‍ ‍μM, respectively. (C) Effects of a 24-h treatment with each reagent on nitrogenase activity. ARA was measured using gas chromatography, and data are expressed as activity nodule^–1^ fresh weight (FW). In ARA measurements, all inhibitors and scavengers were used at a final concentration of 100‍ ‍μM. Data are means±SE (*n*=9). * *P*<0.05 according to the Student’s *t*-test.

The effects of exogenous CSE inhibitors applied to the root system on infection and root nodule formation in *M. loti–L. japonicus* symbiosis were investigated. PAG, BCA, and D-pen were used as CSE inhibitors and NaCN and HT as scavengers of sulfane sulfur and H_2_S, respectively. Three days after germination, seedlings were placed on medium containing the inhibitors and scavengers and were inoculated with DsRed-labeled *M. loti*. PAG and NaCN were used at final concentrations of 50 and 10‍ ‍μM, respectively, because each affected the growth of plants and *M. loti* at concentrations of 100‍ ‍μM (Supplementary Fig. S4A, B, C, and D). Although NaCN inhibits the growth of *M. loti* to some extent, even at 10‍ ‍μM, 10‍ ‍μM was applied for the stability and reproducibility of results. The number of infection threads (Its) was counted 10 days after the inoculation using DsRed-labeled *M. loti*, and the number of nodules was counted at 4 wpi. PAG, BCA, D-pen, NaCN, and HT each reduced the number of ITs and nodules (Fig. 7A and B).

The effects of the inhibitors on nitrogenase activity was also investigated. Whole roots with nodules were soaked in water containing 100‍ ‍μM of each chemical for 24 h, and nitrogenase activity was estimated using acetylene reduction activity (ARA). None of the inhibitors or scavengers significantly affected nitrogenase activity (Fig. 7C).

### Effects of CSE inhibitors on levels of reactive mole­cular species in *L. japonicus* roots

**Fig. 8.**
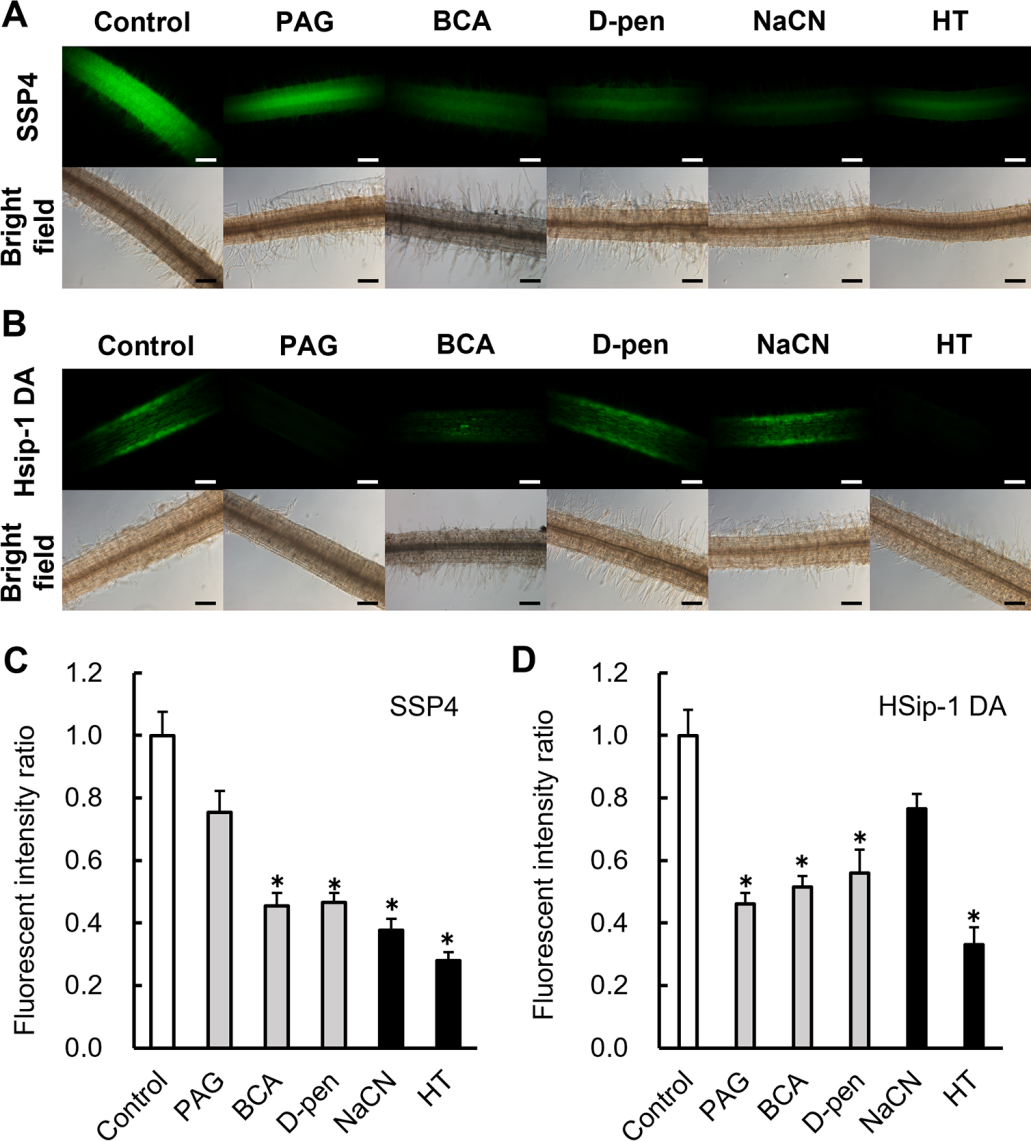
Fluorescent microscopy observations of sulfane sulfur and H_2_S production in roots of *Lotus japonicus*. Seedlings were incubated with PAG, BCA, D-pen, NaCN, and HT for 3 days and then incubated with (A) SSP4 and (B) HSip-1 DA for 1 h. BCA, D-pen, and HT were used at a final concentration of 100‍ ‍μM, while PAG and NaCN were used at final concentrations of 50 and 10‍ ‍μM, respectively. Scale bars: 100‍ ‍μm. Quantification of fluorescence intensity in SSP4 (C) and HSip-1 DA (D) images. In (C and D), values are the means±SE of nine biological replicates, each with three pictures. * *P*<0.05 vs. the control (the Student’s *t*-test).

**Fig. 9.**
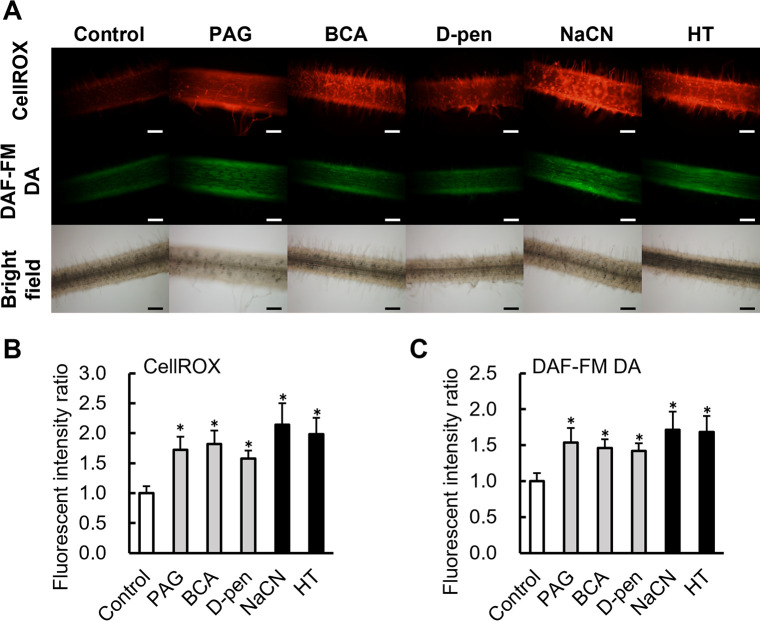
Fluorescent microscopy observations of ROS and NO production in roots of *Lotus japonicus*. (A) Seedlings were incubated with PAG, BCA, D-pen, NaCN, and HT for 3 days and then incubated with CellROX and DAF-FM DA for 1 h. BCA, D-pen, and HT were used at a final concentration of 100‍ ‍μM, while PAG and NaCN were used at final concentrations of 50 and 10‍ ‍μM, respectively. Scale bars: 100‍ ‍μm. Quantification of fluorescence intensity in CellROX (B) and DAF-FM DA (C) images. In (B and C), values are the means±SE of nine biological replicates, each with three pictures. * *P*<0.05 vs. the control (the Student’s *t*-test).

The roots of *L. japonicus* were treated with the CSE inhibitors and scavengers to evaluate their effects on sulfane sulfur and H_2_S levels in the roots. Surface-sterilized seeds were placed on an agar plate supplemented with each inhibitor or scavenger. Three days later, the seedlings were treated with SSP4 and HSip-1 DA, and sulfane sulfur and H_2_S levels were estimated according to fluorescence intensity. Each of the inhibitors and scavengers, except for NaCN, reduced the fluorescence intensity of SSP4 (Fig. 8A and C) and HSip-1 (Fig. 8B and D) and contributed to reductions in sulfane sulfur and H_2_S levels. NaCN did not significantly affect HSip-1 fluorescence intensity (Fig. 8B and D). The effects of the inhibitors and scavengers on ROS and NO levels were also examined. Each of the inhibitors and scavengers increased the fluorescence intensity of CellROX (Fig. 9A and B) and DAF-FM DA (Fig. 9A and C), indicating increased levels of ROS and NO in the roots.

## Discussion

Using *M. loti* with a mutation in its *CSE* gene, we revealed that the CSE of symbiotic rhizobia contributes to symbiotic nitrogen fixation and nodule lifespan via the production of both sulfane sulfur and H_2_S. CSE mutations affected sulfur and amino acid metabolism as well as RSS levels, *e.g.*, cysteine persulfide and glutathione persulfide. Using CSE inhibitors, we revealed that CSE activity in the host plant root was required for infection and the establishment of symbiosis. Furthermore, *CSE* gene mutations in rhizobia affected ROS levels in nodules, while CSE inhibitors affected ROS and RNS levels in roots, indicating that CSE-mediated RSS production contributed to the establishment of successful nodule symbiosis through crosstalk with several reactive mole­cular species.

*CSE* mutations reduced sulfane sulfur and H_2_S production by *M. loti* (Fig. 1A and B), which is consistent with findings reported by Zou *et al.* (2020), namely, that H_2_S production was reduced in *Sinorhizobium fredii* lacking *CSE*. RSS contribute to antioxidant capacity; however, no significant difference was observed in the H_2_O_2_ tolerance of the WT and *cse* mutants under free-living conditions (Fig. 1C and D). In *M. loti*, catalases, such as *katG*, are involved in tolerance to oxidative stress under aerobic free-living conditions (Hanyu *et al.*, 2009). In *Mesorhizobium huakuii*, a gene that functions in glutathione production, *gshB*, is involved in tolerance to oxidative stress (Luo *et al.*, 2020). In *cse* mutants, antioxidant mechanisms, such as KatG and GshB, may contribute to H_2_O_2_ tolerance under free-living conditions.

The relative levels of sulfane sulfur and H_2_S were lower in nodules induced by *cse* mutants than in those induced by WT, indicating that CSE contributes to sulfane sulfur and H_2_S production even under symbiotic conditions (Fig. 5A, B, and C). However, ROS levels were higher in the infected cells of plants inoculated with the *cse* mutants than in those of plants inoculated with WT (Fig. 5A and D). The different effects of CSE on ROS levels under symbiotic (Fig. 5A and D) and free-living (Fig. 1C) conditions may be attributed to the differences in gene expression that occur in different environments. For example, *katE* is associated with antioxidant activity in the free-living state, whereas *katG* is expressed during root nodule symbiosis and contributes to antioxidant activity (Hanyu *et al.*, 2009). A shift between antioxidant mechanisms according to the environment may enhance the contribution of RSS as antioxidants. Increased ROS levels in nodule cells inhibit nitrogenase, but are an essential factor for symbiosis (Santos *et al.*, 2000; Ma *et al.*, 2021). For example, ROS produced by PvRBOHB in *Phaseolus vulgaris* establishes symbiosis through crosstalk with flavonoids, carbon metabolism, cell cycle regulation, and the plant hormones auxin and cytokinin during the early stages of infection thread formation and root nodulation (Fonseca-García *et al.*, 2021). Reduced RSS levels in nodules due to *CSE* mutations may change ROS levels, which may, in turn, affect nitrogen-fixation activity and the lifespan of symbionts.

At 4 wpi, the symbiotic phenotypes of the *cse* mutants did not significantly differ from those of WT, with the exception of nitrogenase activity (Fig. 2). Therefore, CSE activity appears to be required for nitrogenase activity, consistent with symbiosis between soybean and *S. fredii* (Zou *et al.*, 2020), whereas *CSE* mutations exert few effects on the early stages of symbiosis, *e.g.*, during infection and nodulation. At 8 wpi, the growth of plants inoculated with *cse* mutants was significantly lower than that of plants inoculated with WT, suggesting that the CSE of symbiotic rhizobia helps maintain symbiotic nitrogen fixation and is required for long-term symbiosis (Fig. 3). The decrease in nitrogen-fixing activity at 8 wpi lasted longer than that at 4 wpi, which is considered to have had a more pronounced effect on plant growth. CSE catalyzes cystathionine to H_2_S via cysteine (Chiku *et al.*, 2009). In many cases, RSS production is closely related to cysteine and methionine metabolism; however, limited information is currently available on its relationship with other amino acids (Walsh and Giedroc, 2020). We herein demonstrated that cystathionine levels increased while cysteine and H_2_S levels decreased in the nodules induced by the mutants, suggesting that the CSE of *M. loti* exhibited similar activity to that of known mammalian and bacterial CSEs (Fig. 5 and 6). CSE also catalyzes cystine to cysteine persulfide (Ida *et al.*, 2014); therefore, this function may be abnormal in *M. loti* mutants, which may have led to a decrease in RSS production in the nodules. The effects of increased cystathionine levels on overall amino acid metabolism also need to be considered. In *Arabidopsis thaliana*, the accumulation of cystathionine affected methionine and threonine levels (Kim and Leustek, 2000; Amir *et al.*, 2002). The amino acids affected by the *CSE* mutation of *M. loti* also included methionine (Fig. 6D). Therefore, the possibility that increased cystathionine levels affected amino acid metabolism in the nodules cannot be dismissed. Unfortunately, the complex interplay of plant and rhizobial amino acid metabolism within the nodule organ makes it difficult to understand the exact significance of changes in the amounts of each amino acid. However, the present results suggest that the *CSE* mutation also broadly affected overall metabolism because it changed the levels of various amino acids as well as sulfur compounds in the nodules (Fig. 6D). In addition, greater symbiosis breakdown may have occurred in the later stages of symbiosis (8 wpi) than in the early stages (4 wpi) due to increases in the effects of sulfur and amino acid metabolic abnormalities over time.

In the present study, treatments with the CSE inhibitors PAG, BCA and D-pen reduced RSS levels (Fig. 8A and C) and increased ROS and RNS levels in the roots (Fig. 9). Additionally, sulfane sulfur and H_2_S levels were reduced by treatments with NaCN and HT as sulfane sulfur and H_2_S scavengers, respectively. Therefore, CSE activity appeared to affect the production of RSS by both sulfane sulfur and H_2_S, the levels of which affected each other (Fig. 8). We also found that a CSE inhibitor in the rhizosphere of *L. japonicus* inhibited infection and nodulation (Fig. 7A and B). The CSE inhibitor reduced the level of RSS in the rhizosphere (mainly in host plants), indicating that the early‍ ‍stages of symbiosis were significantly affected. The increased levels of ROS and RNS, which function negatively in the early stages of symbiosis when present at excessive levels, suggest that crosstalk among reactive mole­cular species inhibits infection. However, none of the inhibitors or scavengers examined inhibited the nitrogenase activity of *L. japonicus* nodules under the experimental conditions used in the present study (Fig. 7C). In contrast, soybean nodules were shown to exhibit decreased nitrogen-fixation activity after a HT treatment (Zou *et al.*, 2019). Although the reason for this discrepancy is unclear, the concentration of reactive mole­cular species that affect symbiosis may differ depending on the species of plant and‍ ‍rhizobia. For example, in *M. truncatula*-*Sinorhizobium meliloti* symbiosis, treatment with a NO scavenger or an enhancement in the NO scavenging activity of the rhizobia inhibited nodulation (Del Giudice *et al.*, 2011). On the other hand, in *L. japonicus*-*M. loti* symbiosis, enhanced NO removal by the high expression of *LjGlb1-1* did not inhibit nodulation (Shimoda *et al.*, 2009). The reason for this was‍ ‍attributed to slight differences in NO concentrations affecting their respective symbiosis, and similar differences may exist in RSS.

Nodules induced by *cse* mutants and roots treated with CSE inhibitors exhibited reduced levels of H_2_S and sulfane sulfur (Fig. 5 and 8), suggesting that the CSE activities of both the host plant and symbiotic rhizobium function in RSS production. *CSE* of *M. loti*, which did not significantly affect the infection process or nodulation, was involved in nitrogenase activity and the lifespan of the nodule (Fig. 2, 3, and 4). CSE inhibitors in the rhizosphere significantly affected infection and nodulation, but not nitrogenase activity (Fig. 7). Therefore, the CSE activities of both the host plant and its microsymbiont appear to be involved in root nodule symbiosis, but in different manners due to differences in temporality and the site of production. Despite the importance of sulfur nutrition and reactive mole­cular species in root nodule symbiosis between legumes and rhizobia (Anderson and Spencer, 1950; Carter *et al.*, 1980; Krusell *et al.*, 2005; Rubio and Ludden, 2008; Scherer *et al.*, 2008; Varin *et al.*, 2010), these molecules have been characterized independently. The present study showed that sulfur also functions as a reactive mole­cular species in root nodule symbiosis and that both the microsymbiont and its host plant are involved in RSS production. By using rhizobial *cse* mutants, we demonstrated that the *CSE* gene of the microsymbiont is important in the production of RSS. Collectively, the present results provide novel insights into the collaborative function of sulfur and reactive mole­cular species in root nodule symbiosis.

## Citation

Fukudome, M., Ishizaki, H., Shimokawa, Y., Mori, T., Uchi-Fukudome, N., Umnajkitikorn, K., et al. (2023) Reactive Sulfur Species Produced by Cystathionine γ-lyase Function in the Establishment of *Mesorhizobium loti*–*Lotus japonicus* Symbiosis. *Microbes Environ ***38**: ME23021.

https://doi.org/10.1264/jsme2.ME23021

## Supplementary Material

Supplementary Material
