# Supplementary material for: Reactive Sulfur Species Produced by Cystathionine γ-lyase Function in the Establishment of Mesorhizobium loti–Lotus japonicus Symbiosis
Source: Microbes Environ. 2023 Sep 12;38(3):ME23021. doi: 10.1264/jsme2.ME23021 (PMC10522845; doi:10.1264/jsme2.ME23021)
Supplement: Supplementary file 1 — Supplementary Material [file 38_23021_s1.pdf]

Supplementary Fig. 1

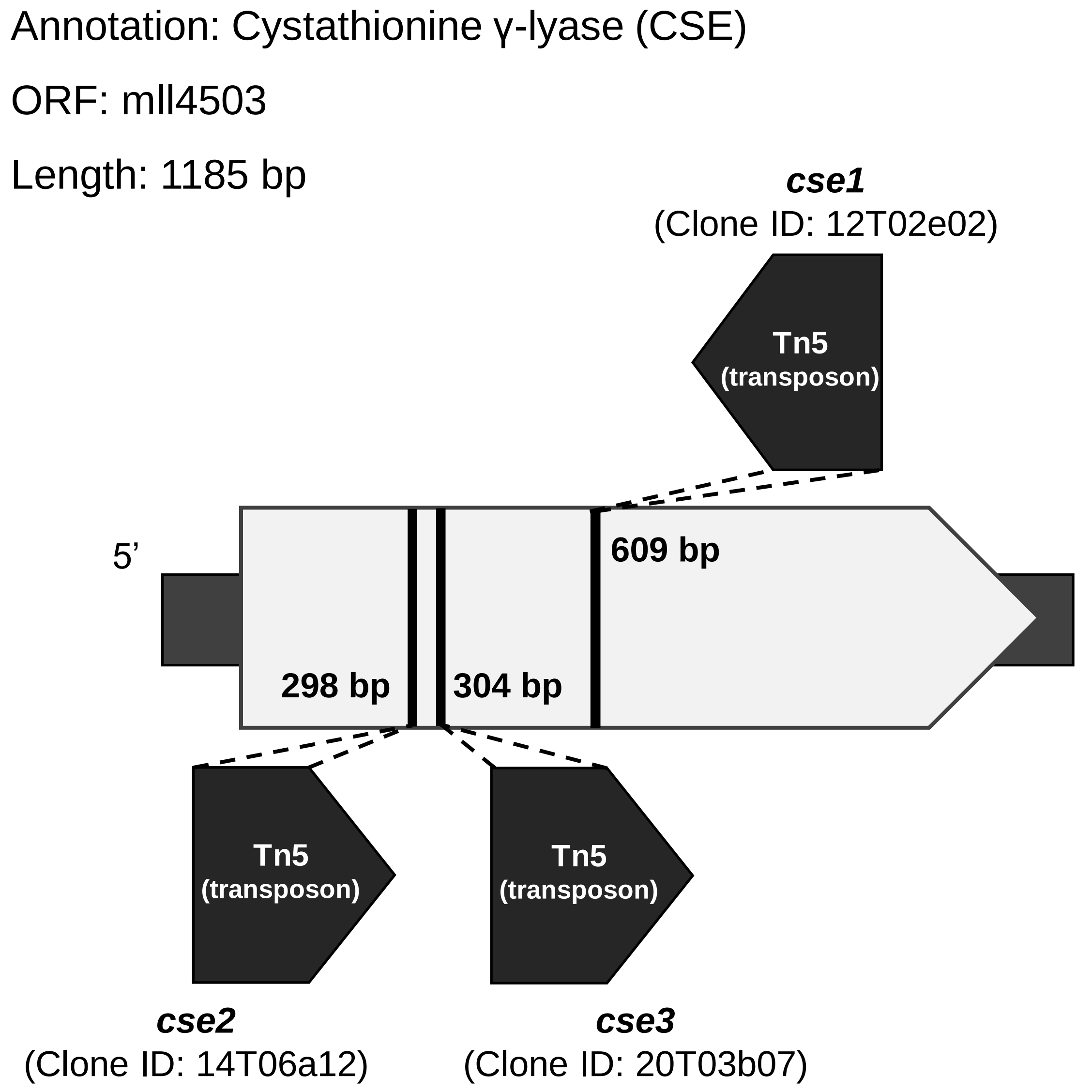

Supplementary Fig. 1 Information on three STM strains with Tn5-based mini-transposons inserted into the ORF of cystathionine γ-lyase (mll4503). Transposon insertion positions for *cse1*, *cse2*, and *cse3* and clone IDs at the National BioResource Project are shown.

Supplementary Fig. 2

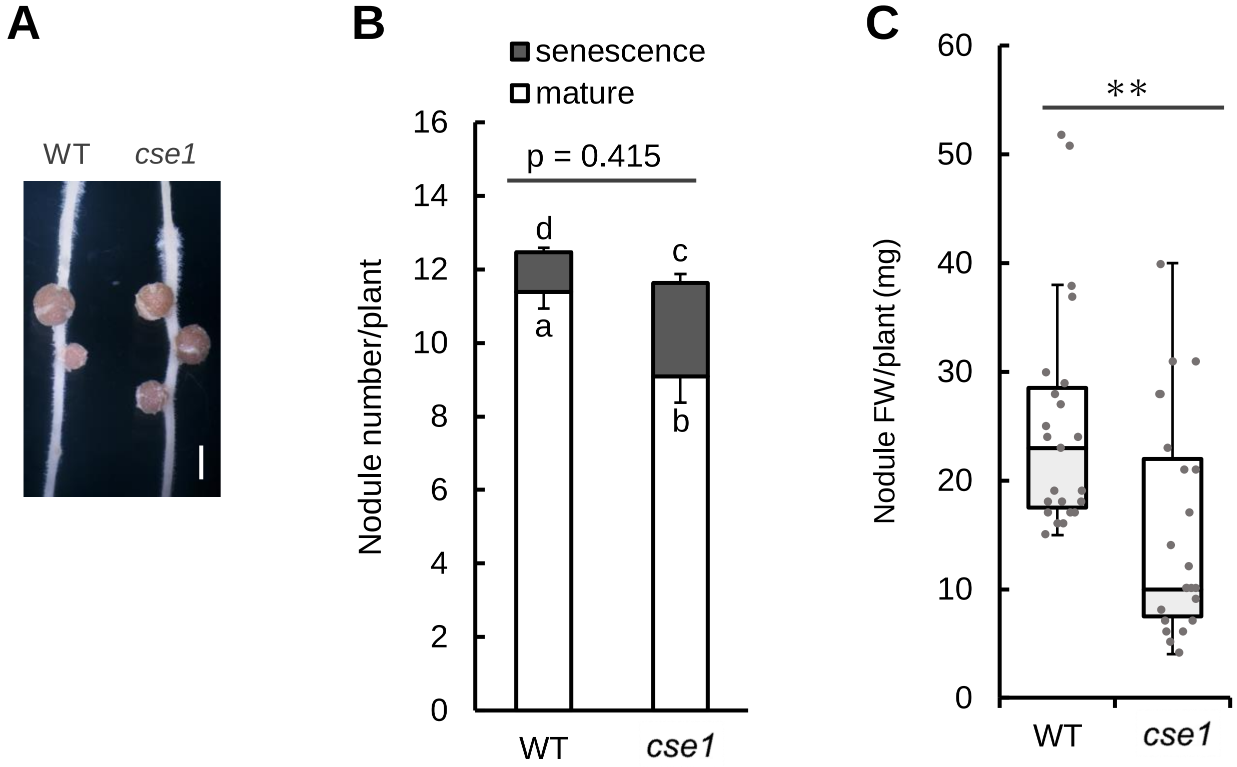

Supplementary Fig. 2 Characterization of the root nodules induced by a *cse* mutant at 4 and 8 weeks postinoculation. (A) Root nodule appearance at 4 wpi. (B) Number of mature and senescent nodules at 8 wpi. White bars indicate the number of mature nodules and gray bars indicate the number of senescent nodules. Data are means ± SE (n = 24). Different letters show significant differences at $p < 0.05$ according to a Tukey–Kramer multiple comparison test. (C) Fresh weight (FW) of total root nodules per plant at 8 wpi (n = 24). ** $p < 0.01$ vs. the control (Student's *t*-tests).

Supplementary Fig. 3

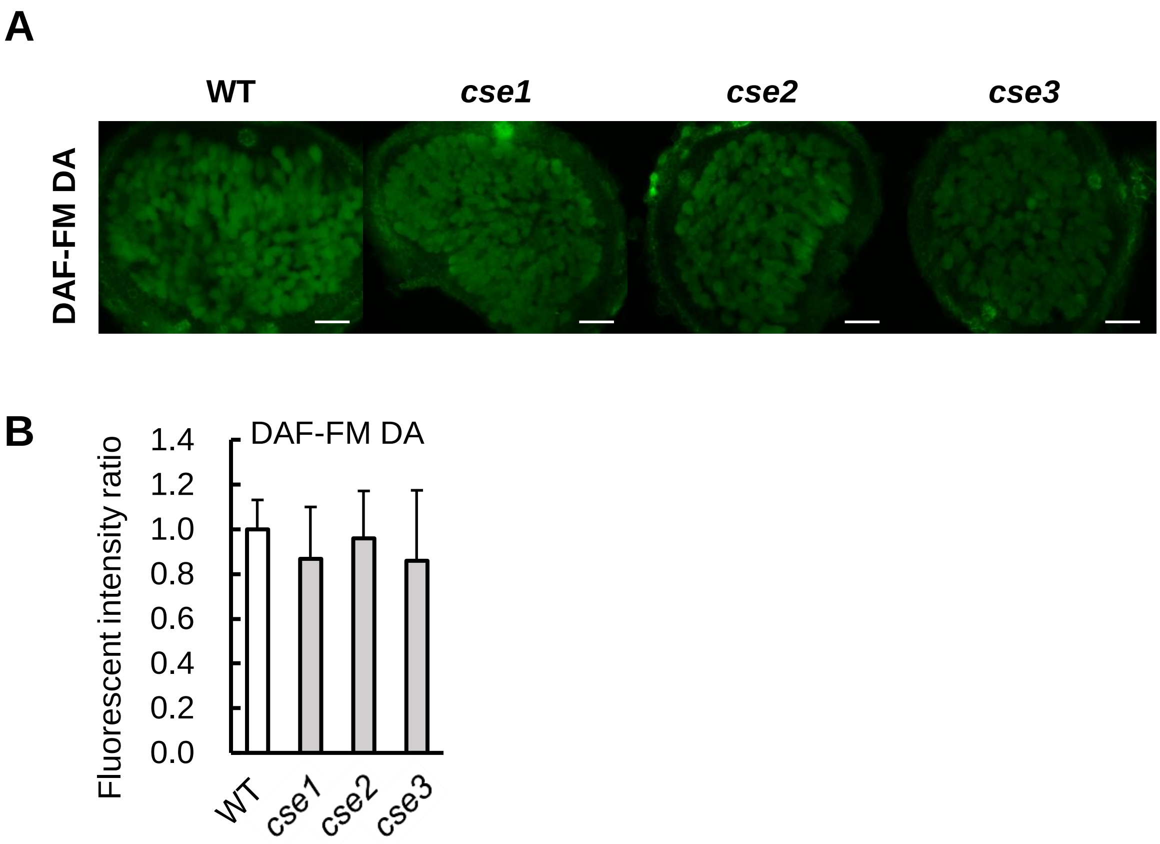

Supplementary Fig. 3 Nitric oxide in the root nodules. (A) Fluorescence microscopy images of WT- or *cse* mutant–infected nodules incubated with DAF-FM DA. Scale bars: 100 μm. (B) Quantification of fluorescence intensity in DAF-FM DA images. Values are means ± SE of nine biological replicates, each with three sections picture.

Supplementary Fig. 4

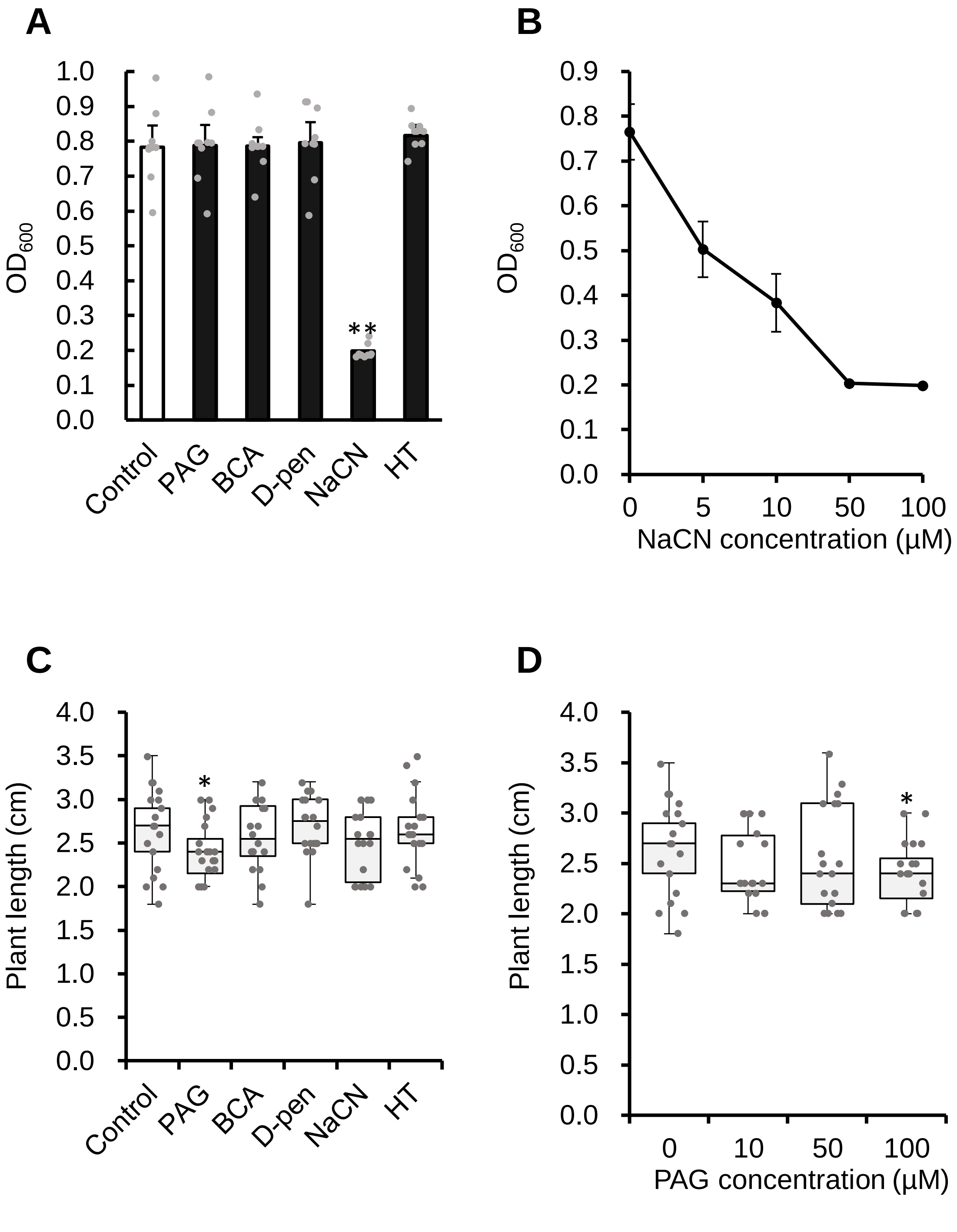

Supplementary Fig. 4 Effects of CSE inhibitors on the growth of *L. japonicus* and *M. loti*. (A) Effects of PAG, BCA, D-pen, NaCN, and HT at a final concentration of 100 µM on the growth of *M. loti*. Cell density at OD_{600} was monitored. Data are means ± SE (n = 9). ** $p < 0.01$ according to Student's *t*-test. (B) Effects of final concentrations of 5, 10, 50, and 100 µM NaCN on the growth of *M. loti*. (C) Effects of PAG, BCA, D-pen, NaCN, and HT at a final concentration of 100 µM on the plant length of germinated *L. japonicus* (n = 18). (D) Effects of final concentrations of 10, 50, and 100 µM PAG on the plant length of germinated *L. japonicus* (n = 18). * $p < 0.05$ vs. the control (Student's *t*-tests).

Supplementary Table 1

Amino acids analyzed via LC–MS/MS.

No.	Amino acid
1	Phenylalanine
2	Tyrosine
3	Methionine
4	Hydroxyproline
5	Isoleucine
6	Leucine
7	Valine
8	Glutamic acid
9	Proline
10	Aspartic acid
11	Threonine
12	Alanine
13	Serine
14	Lysine
15	Ornithine
16	Histidine
17	Arginine
18	Gamma-aminobutyric acid
19	Citrulline
20	Cysteine
21	Tryptophan
22	Homocysteine
23	Cystine
24	Cystathionine
25	Taurine
26	Hypotaurine
27	Glutathione (reduced form; GSH)
28	Glutathione (oxidized form; GSSG)

The 28 amino acids that were simultaneously measurable are listed in the table. Amino acids containing sulfur are marked in gray.

Supplementary Table 2

Sulfur compounds analyzed using the sulfur index method.

No.	Sulfur compound	No.	Sulfur compound
1	Methionine	35	Cys-His-bimane
2	Cys-bimane	36	Cys-Ser-bimane
3	Cys-S-bimane	37	Cys-Thr-bimane
4	GS-bimane	38	Cys-Val-bimane
5	GS-S-bimane	39	Cys-Met-bimane
6	GS-S2-bimane	40	Cys-Ile-bimane
7	GS-S3-bimane	41	Cys-Leu-bimane
8	GS-S4-bimane	42	Cys-Cys-bimane
9	HmCys-bimane	43	Cys-Phe-bimane
10	HmCys-S-bimane	44	Cys-Trp-bimane
11	Sulfite-bimane	45	Cys-Gly-bimane
12	sulfide ion	46	S-Methyl-cysteine
13	Thiosulfate-bimane	47	Taurine
14	Ergothioneine-bimane	48	Cys-Pro-bimane
15	5-Glutamylcysteine-bimane	49	Thiourocanic acid-bimane
16	Cystine	50	Sulforaphane
17	Cystathionine	51	S-Adenosylhomocysteine
18	S-Adenosylmethionine	52	S-Nitrosoglutathione
19	GSSG [M+2H]2+	53	Glucoraphanin
20	S-sulfocysteine	54	Hypotaurine
21	GS-S-SG [M+2H]2+	55	Cysteine sulfinic acid
22	GS-S2-SG [M+2H]2+	56	3-Methyl-2-buten-1-thiol-bimane
23	GS-S3-SG [M+2H]2+	57	L-cysteic acid
24	GS-S4-SG [M+2H]2+	58	Tripanothione disulfide[M+2H]+
25	Lanthionine	59	Tripanothione-bimane[M+2H]+
26	Adenosyl 5'-phosphosulfate	60	Homocystine
27	3'-Phosphoadenosine-5'-phosphosulfate	61	Glucotropaeolin
28	S-Hercynyl-cysteine sulfoxide	62	Sinigrin
29	5-Glutamyl-S-hercynyl-cysteine sulfoxide	63	Gluconapin
30	2-Furanmethanethiol-bimane	64	Phenethyl glucosinolate
31	Benzyl mercaptan-bimane	65	Glucocheirolin
32	4-Mercapto-4-methyl-2-pentanone-bimane	66	Glucoalyssin
33	2-Mercaptoethyl acetate-bimane	67	Glucobarbarin
34	Thiamine	68	S-Lactoylglutathione

Sulfur metabolomic analysis was performed by the Sulfur Index Service in Japan. Of the 68 compounds analyzed, the 28 compounds that could be detected are marked in gray. The "-bimane" in some compounds indicates that they were stabilized by monobromobimane.
